# Living ethics: a stance and its implications in health ethics

**DOI:** 10.1007/s11019-024-10197-9

**Published:** 2024-03-13

**Authors:** Eric Racine, Sophie Ji, Valérie Badro, Aline Bogossian, Claude Julie Bourque, Marie-Ève Bouthillier, Vanessa Chenel, Clara Dallaire, Hubert Doucet, Caroline Favron-Godbout, Marie-Chantal Fortin, Isabelle Ganache, Anne-Sophie Guernon, Marjorie Montreuil, Catherine Olivier, Ariane Quintal, Abdou Simon Senghor, Michèle Stanton-Jean, Joé T. Martineau, Andréanne Talbot, Nathalie Tremblay

**Affiliations:** 1 Pragmatic Health Ethics Research Unit, Institut de recherches cliniques de Montréal (IRCM), Université de Montréal, McGill University, 110 avenue des Pins Ouest, Montréal, QC H2W 1R7 Canada; 2 Pragmatic Health Ethics Research Unit, IRCM, Montréal, QC Canada; 3 CIUSSS du Nord-de-L’Île-de-Montréal, Québec, Canada; 4 School of Social Work, Université de Montréal, Montréal, QC Canada; 5 Sainte-Justine University Hospital, Université de Montréal, Québec, Canada; 6 CISSS de Laval, Université de Montréal, Québec, Canada; 7 CIUSSS de L’Est-de-L’Île-de-Montréal, Québec, Canada; 8 Center of Excellence on Partnership with Patients and the Public, Université de Montréal, Research Centre of the Sainte-Justine University Hospital, Québec, Canada; 9 Université de Montréal, Québec, Canada; 10 Pragmatic Health Ethics Research Unit, IRCM, Université de Montréal, Québec, Canada; 11 CRCHUM, CHUM, Université de Montréal, Québec, Canada; 12 INESSS, Université de Montréal, Québec, Canada; 13 Pragmatic Health Ethics Research Unit, IRCM, University of Oxford, Montréal, QC Canada; 14 Ingram School of Nursing, Centre de recherche de l’Institut universitaire en santé mentale de Montréal, McGill University, Québec, Canada; 15 Pragmatic Health Ethics Research Unit, IRCM, McGill University, Québec, Canada; 16 Centre de recherche en droit public, Université de Montréal, Québec, Canada; 17 HEC Montréal, Québec, Canada; 18 CISSS de Chaudière-Appalaches, Québec, Canada; 19 CIUSSS de l’Estrie-CHUS, Sherbrooke, QC Canada

**Keywords:** Ethics, Health, Living theory, Pragmatism, Dialogue, Collaboration

## Abstract

Moral or ethical questions are vital because they affect our daily lives: what is the best choice we can make, the best action to take in a given situation, and ultimately, the best way to live our lives? Health ethics has contributed to moving ethics toward a more experience-based and user-oriented theoretical and methodological stance but remains in our practice an incomplete lever for human development and flourishing. This context led us to envision and develop the stance of a “living ethics”, described in this inaugural collective and programmatic paper as an effort to consolidate creative collaboration between a wide array of stakeholders. We engaged in a participatory discussion and collective writing process known as instrumentalist concept analysis. This process included initial local consultations, an exploratory literature review, the constitution of a working group of 21 co-authors, and 8 workshops supporting a collaborative thinking and writing process. First, a living ethics designates a stance attentive to human experience and the role played by morality in human existence. Second, a living ethics represents an ongoing effort to interrogate and scrutinize our moral experiences to facilitate adaptation of people and contexts. It promotes the active and inclusive engagement of both individuals and communities in envisioning and enacting scenarios which correspond to their flourishing as authentic ethical agents. Living ethics encourages meaningful participation of stakeholders because moral questions touch deeply upon who we are and who we want to be. We explain various aspects of a living ethics stance, including its theoretical, methodological, and practical implications as well as some barriers to its enactment based on the reflections resulting from the collaborative thinking and writing process.

**Supplementary Information:**

The online version contains supplementary material available at 10.1007/s11019-024-10197-9.

Moral or ethical questions^1^ are vital because they affect our daily lives: what is the best choice we can make, the best action to take in a given situation, and ultimately, the best way to live our lives? Human health and healthcare have long generated situations where moral questions abound and are tied to the very meaning of our lives, of who we are, who we want to be, and how we can contribute to the welfare of others. When unresolved, the impact of these situations is often profound and consequential, leading to existential angst (Kierkegaard 1981 (1844)), moral distress (Jameton 1984), moral injury (Jinkerson 2016) and, more generally, impediments to the ability to question and adapt human practices to foster greater human flourishing (Racine 2024a).

Historically, to effectively tackle moral questions, ethics has transformed progressively from a more theoretical and rationalistic philosophical field common in the early twentieth century, into a more practical, plural, and interdisciplinary field in the last decades. The context of healthcare is one of the chief areas where this change has happened both in theory and in practice. In this context, more deductive stances (see further explanations below on the concept of stances) have been criticized for reflecting an “engineering model of applied ethics” (Caplan 1980), or a “deductive model of ethics” (Hoffmaster 2018). In fact, bioethics scholarship^2^ and practice is credited as historically contributing to moving ethics toward a more experience-based and user-oriented theoretical and methodological stance directly involving patients and healthcare professionals (Toulmin 1982). This is evidenced in the development of clinical ethics support and services as well as empirical bioethics (Siegler et al. 1990; Borry et al. 2005). Moreover, the development of principlism (Beauchamp and Childress 1979), casuistry (Jonsen and Toulmin 1988), virtue ethics (Foot 1978), feminist ethics and care ethics (Noddings 1982; Sherwin 1992), narrative ethics and hermeneutics (Carson 2001; Charon 2001), and the capabilities approach (Sen 1989, 2002; Nussbaum 1997) can all be cited as efforts to embody and enact this more experience-oriented and user-oriented stance. To a different degree, and using sometimes very different concepts, these approaches have attempted to offer an alternative to the rather abstract ethics theory prior to the 1960s and 1970s (Toulmin 1982; Anscombe 1958). They embody a different attitude, a different posture, in brief what we refer to as a stance. Thus, there has been a progression, albeit non-linear and non-uniform, toward envisioning ethics theory as a practical tool of self-understanding and agent empowerment. Of late, movement in this direction has been identified as a progression toward increasingly collaborative dynamics (Rendtorff 2002) expressed as taking the concrete form of facilitation (Walker 1993), dialogue (Widdershoven et al. 2009; Abma and Widdershoven 2014), and moral learning and empowerment through capability-growth (Aiguier and Cobbaut 2016).

Building on these developments and cognizant of the progress made, we convened as a group of stakeholders (e.g., practicing ethicists, patients, healthcare professionals, academic ethicists) to reflect on how we could articulate a stance where even greater commitment could be enacted to the idea of ethics as an effective and accessible lever of human development and human flourishing. We desire not only to support more formal health institutions and their staff but also fellow citizens in a spirit of empowerment and democratization. Thus grew the idea, notably inspired by pragmatist theory, of a living ethics stance. In this inaugural and programmatic discussion paper, we first explain the participatory and collaborative process and methods guiding our work which support the writing of this manuscript (see also section A of online Supplementary Information). Then, we elaborate on the role of stances in ethics from the standpoint of a collective of health stakeholders. Here, we explain the challenges encountered in our ethics work. These challenges ground our collective reflections and our desire to overcome them via the development of a living ethics stance. We distinguish the notion of a stance from common notions such as ethics theory and ethical principles. This explanation mitigates worries that the development of a living ethics stance is an implicit judgment that all other stances are associated with non-living, dead, or dying forms of ethics. After, we explain key features of a living ethics stance as we envision it as well as ideas supporting this notion. These supporting ideas include how a living ethics stance is grounded in an account of moral problems as living problems and also how it builds from pragmatism, living theory and contemporary scholarship (see also section B of online Supplementary Information). We then lay out some of the early theoretical, methodological, and practical implications of this stance as well as some foreseeable barriers to its enactment. The final section is dedicated to potential future development and deployment of a living ethics. Here, we briefly report on initial progress in the early development of this stance and on encouraging new projects and collaborations.

We hope that this paper accomplishes two tasks. First, explaining what a living ethics stance is, at least initially, in this first inaugural and programmatic paper. Second, we hope it instills further regional and international discussions on the different or similar views developed by other groups of colleagues around the world, in their own social and political contexts. We hope that our process and contribution–although perfectible–can serve as an example of how this can be done in a participatory, collegial, and constructive way to engage in more collaborative and communal forms of thinking and writing (Denborough 2008; Gardner 2018; Langley et al. 2018). This effort also aligns with an account of metaethics, i.e., reflection on the fundamental ideas and concepts of ethics, as a “deliberative practice” (Lekan 2006).

## Process and methods

**Fig. 1:**
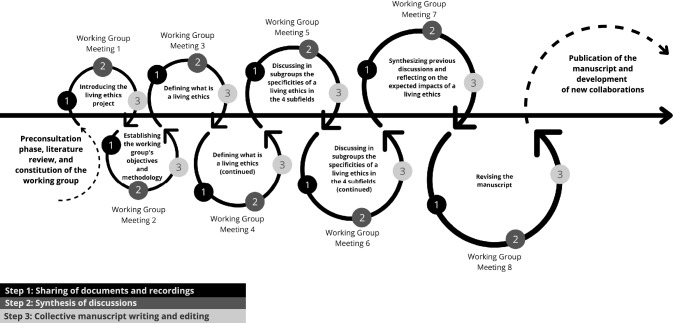
Working group process. Figure was designed using the Canva software

Our discussions and collective thinking and writing followed a collaborative and participatory process method known as instrumentalist concept analysis (Racine et al. 2019). It is comprised of three distinctive steps: (1) function identification, (2) function enrichment, and (3) function testing. In short, this method represents a function-oriented strategy to first critically revisit the tasks of ethical concepts akin to Dickert’s work on consent (Dickert et al. 2017). It then proposes participatory processes to enrich and test for the revised functions attributed to ethical concepts. Detailed methodological information about participants, consultation processes, literature review, working group meetings, and international advisory committee is provided in the supplementary information (see section A of the Supplementary Information). Figure 1 summarizes how the living ethics working group process involved 8 meetings, each structured into three separate steps (Step 1: Sharing of documents and recordings, Step 2: Synthesis of discussions, Step 3: Collective manuscript writing and editing). Our collective work is reported in the following sections starting with an analysis of the function of stances in ethics (Sect. "The Role of stances in ethics from the standpoint of a collective of health stakeholders"), a proposal for enrichment in the form of a living ethics stance (Sect. "Envisioning a living ethics stance"), and discussion about the implications and strategies for testing this stance as well as initial early progress made in this direction (Sects. "Envisioning a living ethics stance" and "Implications of a living ethics stance in health ethics") Thus this entire manuscript should be seen as the result of a collaborative research and writing process.

## The role of stances in ethics from the standpoint of a collective of health stakeholders

Appreciative of continuous and progressive strides in the development of more engaged stances in ethics, we–as a group of authors active in multiple areas of health and health ethics in various disciplines and professions in the province of Québec, Canada,^3^ – make a few key foregrounding self-critical observations about the functions served by a user-centered and collaborative stance. Indeed, despite the multi-faceted movements toward more situated, substantive, and practical forms of ethics we observe that our individual and collective work in ethics remains an incomplete lever for human development and flourishing. This can be seen in various ways, but we share here three such illustrative instances grounded in our own work and experiences.^4^

First, we–like members of other health ethics networks worldwide–use and have participated in the development of methods and approaches (e.g., Doucet scenario-based method as described in (Baertschi 1998); moral case deliberation (Stolper, Molewijk et al. 2016)) moving beyond norm compliance to engage with deeper habits and ways of being, of thinking and of doing. However, there is still undoubtedly considerable demand for ethics conformity (e.g., in the form of professional codes of conduct, regulatory research ethics) and corresponding uses of ethics to instill corporate compliance (Craze 2020). Ethics interventions are often caught in minimalist understandings of ethics and in authoritative expectations toward ethicists while the aim of ethics is to do and offer more (Inguaggiato et al. 2019). Public decision-makers who seek support from ethicists sometimes struggle to understand the nature of ethics, which makes it hard to explain to them why ethics matters and why ethics should be part of important public policies (Brennan, English et al. 2021). This demand for compliance is often a shock to how we envision and want to enact ethics scholarship and practice following a user-centered and collaborative approach. But the need for compliance is a complex situation attributable partly to how ethics scholarship is mobilized and is connected (or not) to the experience and words used by protagonists encountering moral problems (see epigraphs by Peirce and Callahan). In other words, there is an ongoing need to connect ethics scholarship to substantive experiential and existential problems that give meaningfulness to ethics beyond compliance and norm-following. Common assumptions about what ethics is, and is not, prevent more meaningful engagement with substantive goals of ethics because the stance of a collaborative and user-centered ethics is not well and commonly appreciated.

Second, as a collective of interdisciplinary colleagues (including several who are also patient-partners), we struggle to break the boundaries between the various subdomains of ethics and to develop collaborations based on a shared vision around an ethics stance. In other words, there is a need and desire to decompartmentalize ethics in the coauthor group–even much beyond healthcare–to create bridges between economic, environmental, political, and educational considerations. There is a creeping form of digital bureaucratization in public and health services due to the increasing place of computerized systems and the use of artificial intelligence instead of real people (Deloitte Centre for Health Solutions 2020). As Newman and colleagues write, bureaucracy is not disappearing, it is being empowered and enabled by computerized technology, including in healthcare and social services (Newman et al. 2022). Although not all negative and not well understood, these changes currently generate concerns since they may limit the ability to exchange in-depth on ethical problems, as implied by a user-centered and collaborative stance, when discussions do not fit pre-established categories, divisions, and systems. There is thus perhaps, more than ever, a need to create times and spaces where ethical discussions can truly happen (Walker 1993), and in ways that allow broader, relational, and more holistic thinking in the spirit of initiatives aimed toward sustainable health, global health or “one health” (Lang and Rayner 2012; Capps 2022). To generate such spaces in ways that tap into different domains, there would be a benefit in agreeing and sharing around a stance which would bring the added value of breaking silos to develop ethics as a more effective lever of human development and flourishing.

Third, and perhaps most importantly, we observe that fellow citizens remain frequently estranged from ethics to a degree where ethics is often regrettably understood as a domain of expertise with abstract or intractable questions for which there are moral experts and ethicists, who answers these questions for everyone. Although formal ethics services intend to help patients and families, the latter often know little about them (DuVal 2001, Neitzke 2009). Ethics theory is often geared toward tooling healthcare professionals, as seen in some of the most influential work in the field (e.g., principles of biomedical ethics to guide healthcare professionals and biomedical scientists (Beauchamp and Childress 1979) or casuistry to guide clinicians (Jonsen et al. 2010)). In our view, these perceptions and practices limit our interwoven current practices and understandings of ethics, and have direct implications for our respective domains of work, as notably also reported by others internationally (Andre 2004, Iltis, 2016). There is thus a need for more accessible forms of ethics interventions and processes to allow fellow citizens to engage with ethical questions and participate in deliberations. Defining the contours of a stance that would make this easier and explain its value could serve to facilitate access to ethics support and services, perhaps even leading to new forms of services which are more accessible and more user oriented.

These three observations led us to want to reflect openly as a collective on the implicit assumptions and orientations guiding our thinking and our practices, and then envision and develop a stance from which ethics can become a more profound, complete, and accessible lever for human development and flourishing. Building on the historical movement toward more substantive, engaged, and embodied forms of ethics theory and practice, we aim to operationalize greater synergies between ethics theory and ethics practice and experience, by embarking in the project as a collective of interdisciplinary colleagues. Thus, we see the initial task of further envisioning a *living ethics* as a way to progress toward the development of ethics as an effective tool of human development and flourishing in the context of health. We described this task as “elaborating, defining and enriching, together, the idea of the stance of a living ethics in order to clarify the meaning of this idea and related practices and concepts, and to outline its methodological orientations, and its foreseeable practical outcomes”. Undertaking this task does not mean that ideas associated with a living ethics stance were not and are not present in our (and for that matter others’) past and current work, but rather that a living ethics stance embodies a vision of what we aspire to for our work and contributions. Excavating this vision and making it explicit is a promise for greater collaboration and shared sense of purpose.

This manuscript reports on the initial description and implications of the idea of a living ethics stance.^5^ We formulate this collective reflection under the banner of a “stance” rather than a concept, a theory, or a principle because the response to the kinds of challenges and shortcomings described above did not appear to us as residing in the production of new theories. Rather, they appear to lay in the orientation toward the use of theories, i.e., to paraphrase Montesquieu, the spirit guiding the use of theories and various practices (Montesquieu 1979 (1748)).^6^ We came to the view that we needed to clarify a stance to describe how we desire–in response to the above observations: (1) to connect ethics scholarship to substantive experiential and existential problems that give meaningfulness to ethics beyond compliance and norm-following; (2) to create times and spaces where ethical discussions about such problems can truly happen; and (3) to facilitate more accessible forms of ethics processes to allow fellow citizens to engage with ethical questions, and participate actively in deliberations.

An explanatory remark is in order about the idea of a “stance” in the context of ethics in order to disambiguate it from other common notions such as ethical principles, ethical theories, and ethical concepts.^7^ A stance could be defined as a position or posture taken within a given context or, alternatively, as a vantage point from which knowledge is generated and used and from which practices make sense (or not). This idea is somewhat analogous to Daniel Dennett’s deployment of this concept in philosophy of mind to describe different perspectives and related explanatory strategies (Dennett 1981).^8^ A stance could be characterized in different ways, such as being practical or theoretical, engaged or disengaged, resolute or indecisive. Although a stance often means that a certain commitment is adopted or a position is deliberately taken, a stance can also be implicit within practices. Indeed, as “perspectives or ways of seeing” (Boucher 2014), stances often reflect pre-theoretical assumptions embedded in values and practices. They can be easily overseen and not explicitly articulated or debated.

A stance is not a theory since it does not offer an explanation about realities or a comprehensive set of claims explaining (for explanatory theories) or guiding (for normative theories) a phenomenon or a process. However, a stance orients the use of theories toward certain ends and goals. It resembles the notion of a standpoint encountered in feminist standpoint theory although in this case, there are additional aspects related to how people in marginalized social positions develop specific standpoints and epistemological insights (Bowell 2023). Accordingly, a stance can be compatible with aspects of several theories, because ideas and meanings are embedded in more basic–often implicit–assumptions which can be shared across theories following scholarship about implicit and embodied epistemologies and knowledge (Gallagher 2005; Gibson 1979; Varela et al. 1991; Underwood 1996). Accordingly, a living ethics stance is not in competition with, for example, narrative ethics or dialogical ethics, but it represents a standpoint from which these can be considered and used. A stance is not an ethical principle which can serve as a specific benchmark to guide and evaluate practices, but it presents a more basic orientation and set of assumptions about how ethical principles can be used (e.g., more universally or more contextually; more deductively or more collaboratively). Again, the direction indicated by various ethical principles can make sense from a given stance since a stance is the spirit or mindset according to which the principles will be used. Because it represents a mindset, a stance is not equivalent to sectorial descriptions of ethics (e.g., “ethics in practice”, “bioethics” as an ethics of biomedical science and health care, when these words do not represent a specific orientation) because a living ethics stance does not designate domains of application of ethics, but an orientation toward those domains and the process of enactment of ethics. Although scholarly activity can be discrete about what kind of stance it takes, concrete action and activity can more difficultly escape being situated and adopting implicitly or explicitly some stance. From the standpoint of an authors’ collective desire to reflect on what ethics should be and wanting to take action within a specific society, the assumption of a stance cannot be truly avoided.

Sharing a stance may stimulate the creation of communities where colleagues share deeper and clearer assumptions about the nature and goal of their work. At the same time, discussions about stances can be very divisive because they touch upon less theorized habits which become embedded assumptions embodied in practices. The recognition that knowledge and practices are situated and embedded in relationships is key to the idea of stances and of their importance as drivers of knowledge production and human practices. This recognition is a legacy of successive contributions of notably, historical materialist (Marx 1959), pragmatist (Dewey 1922, Mead 1934 (2015)), and feminist (de Beauvoir 1947/2011) scholarship which have all explained in different ways and illustrated compellingly how our own social situations shape goals, values, and interests in each of us. In many respects, the notion of a living ethics stance builds on ideas that pragmatism represents an attitude (Martela 2015) and in our case a democratic and social attitude insofar as it is developed and shared by various stakeholders.

## Envisioning a living ethics stance

### Moral problems as living problems

The development of a living ethics stance starts from the observation that moral problems are living and vital problems. Moral problems are living problems in so far as they are deeply connected to our multifaceted (e.g., professional, personal, public) embodied and relational lives. This is why moral problems cannot be tossed aside: human beings are guided by implicit or explicit interests and values which profoundly shape their identities (Hitlin 2003; McDonald 2011). Accordingly, whenever value conflicts are ignored, there is a risk of generating significant discomfort, moral distress, and even moral injury, which all signal to a different degree the inability to actualize who we are and what we consider important in particular situations (Jameton 1984; Litz et al. 2009; Jinkerson 2016). Similarly, moral distress and moral perplexities are connected to our identities as persons because moral affairs touch upon what we find most significant and intrinsically motivating (Deci and Ryan 1985; Ryan and Deci 2000; Morley et al. 2019; Tigard 2019). When recognized, moral distress and moral perplexities can be tackled to enact what is considered valuable (Rushton 2016).

Moral problems are also alive in the sense that they are part of dynamic human experiences, shaped by social contexts, and evolving as we experience them, think about them, and tackle them. Importantly, for each person occupying a situated position in the world, the meaning of an experience and what is at stake will vary, largely because each person lives a unique life from a unique standpoint from which life events are experienced and evaluated. Taking into account this existential and personal rooting of moral questions is challenging, yet unavoidable, because individuals evolve in various social and professional roles as well as in social systems which influence the ability to express and enact the values at the foundation of their identity and integrity. Importantly, ethics itself has often been envisioned as the search for general norms and values (e.g., codes of ethics) where the enactment of what matters to persons is unclear since norms and values are often imposed in ways that leave limited room for reflection and discussion (Hoffmaster and Hooker 2009; Hoffmaster 2018; Racine 2019). This disconnection between ethics and lived experience can harm people as noted by feminist, pragmatist, and anti-colonial scholars (Azikiwe 1931; Walker 2003; Fiester 2015; Racine 2016). A living ethics stance could empower stakeholders to initiate such a reconnection.

### A living ethics stance

**Fig. 2:**
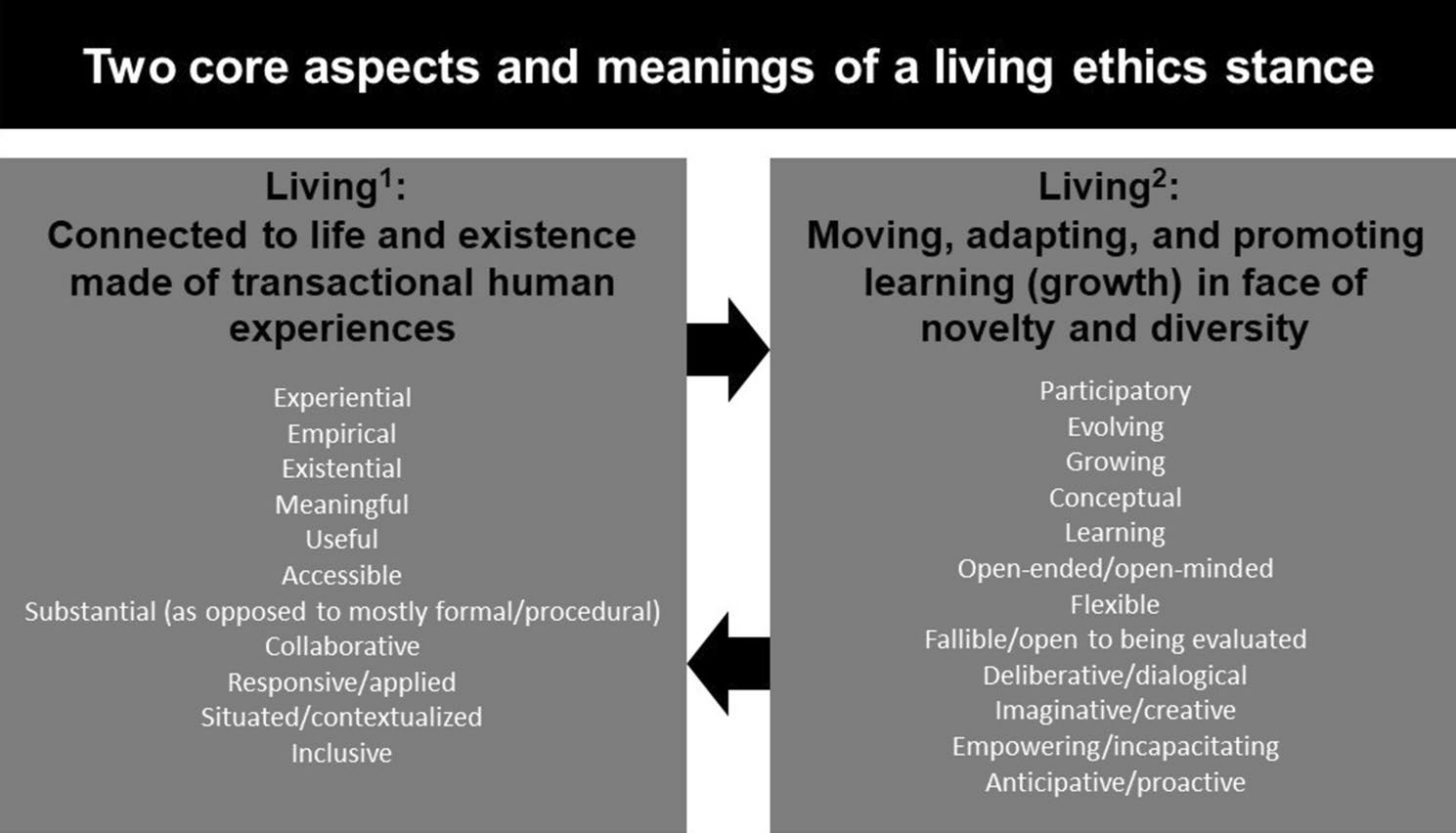
Two general aspects of a living ethics stance and their key characteristics. Figure was designed using the Canva software

A living ethics stance can be unpacked based on two fundamental understandings of what living can mean (see Fig. 2) in relationship to the two aspects of moral problems as living problems described above. In its first sense, living ethics designates a stance deeply preoccupied by human experience and the role played by morality in human existence. In this sense, it calls for life philosophies, ways of making sense of the moral aspects of human existence and of pursuing a moral and meaningful life, akin to how ancient ethical philosophies where developed and practiced (Hadot 2002; Parry 2021). Too often, the language used in ethics is abstracted to the point of reductionism such that important problems are reduced to words (Callahan 1973). Or the language of ethics can verse into rationalism, thus evacuating substantive preoccupations (i.e., “thick” descriptions or experience-near descriptions) because of an over-riding preoccupation with the rigor of moral reasoning which can lead to thin descriptions or experience-distant descriptions (Geertz 1973, 1974). Fiester (2015) has explained how such reductionism in clinical ethics consultations alienates people from their actual moral experiences. Thus a living ethics stance must thus ask how moral language such as principles or values describe meaningfully and truthfully what people live and desire to live. Accordingly, a living ethics stance fits with an understanding of ethics as an experiential, existentially meaningful, empirical, usable and approachable to non-experts, collaborative enterprise (Fig. 2). It encourages being open to understanding experiences from the point of view of those concerned and thus must be profoundly situated and inclusive (see Fig. 2). A living ethics stance therefore implies that we must remain mindful of how our actions (e.g., in terms of ethics intervention, investigation, or awareness-raising) concretely connect to human experience and, eventually, can be brought back–as a result of ethics inquiry–to enrich human experience in helpful ways (Dasgupta, Lockwood Estrin et al. 2022). Living ethics represents a stance toward moral existence and an expectation toward ethics, akin to what Hadot described as being the stance of the ancient Greek and Roman thinkers toward philosophy, namely as an embodied and embedded way of life (Hadot 2002). However, as we describe below, there is much to improve to foster greater connections of ethics to lived moral experiences (Hunt and Carnevale 2011) and to use knowledge about human morality to support human flourishing as shown in ongoing debates about the use of empirical research in ethics (Davies, Ives et al. 2015).

In its second sense, a living ethics stance represents a position from which there is an ongoing effort to interrogate and scrutinize moral experiences, with a special emphasis on the difficult and problematic ones (e.g., dilemmas, difficult choices), in order to promote engagement (e.g., of individuals, teams, institutions, communities). In this engagement, stakeholders are encouraged to envision and enact scenarios anchored in authentic and meaningful goals and values. Accordingly, if “the future belongs to those who believe in the beauty of their dreams,” as pronounced by Eleanor Roosevelt (Shapiro and Epstein 2006), ethics is a process through which our dreams and our visions of our futures are beautified and enriched in terms of meaningfulness including existential and experiential meaning. In other words, ethics ambitions to improve our understanding of what it means to be human and to support human beings in adapting, evolving, and progressing individually and collectively while keeping an open disposition toward others. Perhaps more importantly, we can learn from each other about what matters in order to live a good life and this represents an avenue for reconstructing views based on open deliberation (Callahan 2005) (See also Chapter 6; (Racine 2010)). This openness of a living ethics stance implies being flexible and open to testing the fallibility of ideas guiding our approaches and being willing to deliberate openly and respectfully about difficult human experiences and think about possible action scenarios that can help surmount our difficult lived situations.

“Living ethics” is a radically participatory, grounded, and situated stance. By radical, we here mean that the very essence, the root (*radix*) of ethics, calls for a fundamentally dialogical–or what the pragmatists call a transactional^9^ view of human existence (Brinkmann 2011)–which stresses the importance of relationality and the social self (Mead 1934 (2015)) (see also for a similar view Taylor (1991)) as key aspects of who we are as human beings. A living ethics stance is therefore one where ethics is never entirely accomplished since it is always open to learning and challenging, thus revisiting strong claims to universality or objectivity in order to maintain the connection between scholarly work and everyday life experience. Prospective and future-oriented thinking as well as open-mindedness are essential to a living ethics stance by which questions are asked about the value of human experience and about which kinds of life experiences could and should be pursued in contexts such as clinical care and health organizations, health policy and public health as well as biomedical research and technology. The view that moral problems are living, everyday problems, with existential implications has resonance with several traditions of scholarship (e.g., hermeneutics, narrative ethics, feminist ethics), which we acknowledge and cannot exhaustively review here (see section B of the Supplementary Information).

## Implications of a living ethics stance in health ethics

A living ethics stance has potential implications for ethics and its deployment in multiple sectors of human activity (e.g., business, health, education, governance, environment, law). Our group was constituted to ponder the implications of this stance in health and health ethics. Accordingly, in the following paragraphs, we detail some of the–coarsely divided–theoretical, methodological, and practical implications of adopting a living ethics stance in this context.

### Theoretical implications

#### Ethics theorizing as a personal existential exercise

We realized that our own process of thinking and reflecting on the nature of what a living ethics stance is could not spare us from individually envisioning ourselves as moral agents and determining what a living ethics means for us as individuals. A living ethics stance brings to the forefront that moral issues are questions which affect us as persons; they concern the sense of who we are, notably our identities and the values that make us who we are (see also for a similar view on this point (Hitlin 2003)). Firmly grounding this connection between the first person and the third person helps recognize the intersubjective nature of ethics because, from the very words used to express deeply personal moral anxieties to the actual experience of solutions to moral problems, ethical processes are never purely subjective nor purely objective; they are intersubjective processes. Therefore, exercises in living ethics require openness and creativity to launch sometimes difficult conversations between people as moral agents.^10^ A living ethics stance invites a transition from ‘doing ethics *for* others’ as a detached service where one can teach unilaterally from their perspective to ‘doing ethics *with* others’ such that starting from where people are making co-learning part of the very fabric of ethics itself (Doucet 2006). In fact, a living ethics stance is not owned by anyone or any specialty, discipline, or institution, but is something which takes place and occurs with and within people such that in principle a living form of ethics is relevant to everyone with no one excluded, thus calling for a genuine collaborative spirit. This collaborative approach could in fact facilitate inclusive and globally integrated ethics interventions, e.g., in healthcare settings or in research, by helping to identify commonalities across diverse contexts.

#### The spontaneity of moral life

Another theoretical implication of a living ethics stance is the recognition that moral aspects of human existence are pervasive because values and interests–from the more basic ones, such as simply living, to the more complex ones, such as living a meaningful life–are part of each moment of human existence (Zizzo et al. 2016). A living ethics therefore needs to provide for the fact that moral conflicts and tensions can occur at anytime, anywhere, about any topic, and involving anyone. Thus, ethics can be called upon to address all aspects of personal, interpersonal, and social life, which means that discussing moral concerns can be confronting and uncomfortable by the very nature of what is at stake. Indeed, ethical questions can be raised spontaneously and without deference to conventions and norms. Although morality can live in the implicit and the tacit, ethics is an attempt to name, make explicit, and discuss human morality openly. Accordingly, ethics can be personally demanding because it calls upon the coherence and integrity of who we are in various roles in life, notably asking difficult questions about our own coherence and those of our social, professional, and political networks. The ability to recognize this and to give ethics space and time allows ethics to live and connect to real life situations. In fact, ethics conversations and ethics learning is facilitated when spaces and time are granted (e.g., in work, professional, interpersonal contexts) to the expression of moral difficulties and to their discussion. This possibility is challenged when ethics is amounted to law and stifled by the fear of lawsuits (Walker 1993). When no such spaces exist, and even when they do, ethics often disrupts established practices because it brings to light tensions, and questions the practices leading to these tensions.

#### Dialogue

Given the transactional nature of ethics in the pragmatist sense (Brinkmann 2011; Foucart 2012/3), a living ethics stance calls for the recognition of the dialogical relationship between oneself and others such that it is recognized that a person is constituted by relationships with their environment and with others (Taylor 1991; Crick and Bodie 2016). When this is recognized and acted on, moral existence can be envisioned constructively as an open-ended dialogical process where constant transactions with others helps to reflect on and expand one’s ethical outlook through listening, talking, and learning. Conversations are transactional terrains where such discussions are triggered, to recall the introductory citation from Peirce. Ethics interventions are thus very often dialogical interventions by nature as they help us understand through words and meanings how we are confronted with moral difficulties and how to find and share common language meaning to surmount them.

#### Epistemic humility

Finally, a living ethics stance calls for epistemic humility where experienced moral problems and corresponding responses are first seen through their situated nature by focusing on the context of health and healthcare settings. This does not mean that questions about whether certain given problems and responses cannot be defended in the light of values and principles (e.g., autonomy, justice, equity, diversity). It simply means that the embedded and embodied nature of these problems and questions can be disserved if the contextual nature of these is neglected or discounted by concerns to universalize peculiar understandings of problems and specific responses to them. Consequently, our initial proposal for a living ethics stance is neither prescriptive nor universal and emerges from our situated position as authors coming from various health domains and other domains like education and policy making.

### Methodological implications

#### Methods capturing movement and supporting creativity

A living ethics stance brings about methodological orientations in line with its theoretical inclinations. A living ethics stance in the context of health calls for methods which are evolving, creative, adaptive, and moving to reflect on the evolving and learning nature of ethics as well as understandings of health and illnesses as well as the ongoing changes brought into healthcare sciences and healthcare practices. Much has been written to support the adaptive, prospective, and engaged nature of ethics but these approaches need to be operationalized in various areas of bioethics more radically. In this regard, participatory approaches represent a promising avenue which does not necessarily displace more traditional object-subject epistemologies, but rather bring forth fully the intersubjective nature of human experience and human knowledge (Abma et al. 2017, Montreuil, Thibeault et al. 2017, Cascio 2019, Cascio et al. 2020). Indeed, participatory approaches propose that the more fundamental perspective to adopt is not that of the spectator but that of the actor, i.e., the person engaging with the moral dimension of life and using ethics to find a way of moving forward to more encompassing and inclusive horizons for human experience (Kestenbaum 1992). This is a fitting methodological orientation for ethics if ethics is meant to empower human beings and their moral lives to live their own lives as a life worth living (Racine 2024b).

#### Democratic methods and pluralism

To empower everyone involved in difficult situations or complex discussions, a living ethics espouses a strong commitment to methodological aspects and implications of pluralism and democracy. These are not valued for formal or regulatory reasons, but because they reflect that ethics represents a project of empowering each person with respect to thinking about the meaning of their own life and of the impact of their actions, akin to the pragmatist idea of democracy as a way of life (Pappas 2008). As this is done, pluralism of moral values is an inescapable reality linked to each and everyone’s different experience of the world. Ethics’ alignment with democracy as a deliberative practice reflects that it is a project of living a worthy individual and collective life. As such, if this applies to all, then ethics must build methodologically from moral pluralism and thus work out fitting arrangements based on open processes. In other words, democracy represents a way of governing but also a way of living collectively, where discussions are premised on the consideration of each person, such that together, people can move potentially from forms of chaotic pluralism to agreements on what matters and is beneficial (Pappas 2008). The open and democratic posture of living ethics does not amount to relativism or nihilism since it does not abdicate the need to account for ethical positions even if it takes its distances from the applied ethics “engineering model” of ethics (Caplan 1980).

#### Eudaimonia and substantive questions

*Processes* are important to ensure transparent and democratic approaches in ethics, but this does not neglect that ethical questions are substantive questions about the good life. Several accounts of ethics have tended to thin-down ethics to its procedures and formal aspects of institutions (e.g., theories of justice, discourse ethics) while these make most sense insofar as they contribute to actual goods and the actual possibility of people to pursue good and flourishing lives. A living ethics stance brings back to the methodological forefront the substantive issues that often cause uneasiness and prompt the need for ethics inquiries in the first place. This being said, fair and just processes are essential to support these discussions such that both the ends (substance) and the means (process) are kept in sight. Although these later points may sound like human flourishing brings an *individualistic* focus to ethics, the vast majority of human beings–as reflected in contemporary accounts of wellbeing and flourishing–see human flourishing in light of one’s contribution to others (VanderWeele 2017; Cele et al. 2021). Accordingly, a living ethics stance justifies great methodological attention to human relationships. It reflects what has been learned on the importance of the social self (Mead 1934 (2015)) and relational and contextual autonomy (Mackenzie and Stoljar 2000, Racine, Kusch et al. 2021) because of the importance of relationships in understanding substantively the nature of ethical questions.

#### Self-reflection

Sustaining self-reflection is another methodological orientation congruent with a living ethics stance because self-reflection is a way to take ownership of one’s moral life, of one’s development as a moral person and to cultivate curiosity, humility, and engagement about moral aspects of social and personal life. Reflexivity and self-reflexivity are ongoing processes that have relevance at all stages of ethical inquiries and in the different roles that ethics and ethicists play in the situations in which they intervene (Racine et al. 2017). Reflexivity can be seen as inherent to ethics, but it is also disruptive because it raises questions about the coherence and integrity of moral agents, notably between their activities in different spheres of life. The stance of living ethics cannot be that of moral conformity and compliance which, as we described above, are often conflated with ethics and have stiffened ethics. Rather, it must entice the creative and imaginative mobilization of practical approaches which tap into lived experience and connect to life narratives.

### Practical implications

#### Increasing discursive capacity and opening communication

A living ethics stance leads to numerous orientations for greater practical engagement with moral matters. Increasing discursive capacity and communication about moral aspects of health and human life is paramount to living ethics because ethics can hardly live without the ability to voice moral matters and to discuss them. In the healthcare settings we evolve in, finding time and space for this can be a barrier as health issues per se can be sensitive topics of discussion in pressured environments. Moreover, people vary in their ability to communicate and the ways they prefer to do so. Thus, a living ethics stance favors growth in communicative and discourse capacities–including the actual contexts–and various ways of increasing these (e.g., through the arts and other forms of expression) are of primary importance. Projects rendering ethics more accessible (e.g., through the promotion of public dialogue and participation) are consistent with the user-orientation of a living ethics (Jennings 1990, 2022; Jennings et al. 2021). A living ethics stance is also congruent with efforts to express the many perspectives and many ways people make sense of experience because each person experiences situations differently. It also follows suit with efforts to include in ethical discussions stakeholders from different horizons (e.g., domain of activity, socioeconomic status, levels of education, differences in values). Additionally, because ethics concerns everyone, practical interventions that make ethics more accessible and understandable (e.g., knowledge transfer, public education, awareness-raising) resonate with a living ethics stance. Likewise, awareness-raising educational interventions and methods that follow horizontal teaching methods are ideal ways to prepare children and youth for the active participation in their health and healthcare (Carnevale, Collin-Vézina et al. 2020), and in a democratic citizenship with respect to health and health issues.

#### Incorporating relationality and narrativity

Another important practical orientation is related to relationality and narrativity because what is morally experienced is often best accounted for in narrative form, i.e., as a story of one’s personal trajectory or as a meaningful episode of life (Carson 2001; Charon 2001). Although some forms of expression may be–or appear as–non-narrative, they will need to be interpreted in the context of someone’s life to be made sense of. Thus, *living libraries* of moral experiences and testimonies (Little, Nemutlu et al. 2011) and other platforms of exchanging and relating moral experiences in more narrative format are in keeping with a living ethics stance. In research projects, *research diaries* are a specific method which can be used to help participants report experiences narratively (Bolger et al. 2003). Likewise *living documents* (e.g., participatory position papers) seen as ongoing projects of reflection and writing, and that are open-ended, inclusive and participative are other ways of fostering exchanges and iterative thinking (Gardner 2018). *Field projects* where observations, exposure, and participation in various contexts is encouraged is also in line with a living stance to ethics because of the ability to learn by being exposed to the situations in which others evolve with their corresponding needs (Hoffmaster 1992; Montreuil and Carnevale 2018).* Methodologies that grasp lived experiences (*e.g., narrative-oriented approaches and methods) and *empower action* (e.g., participatory research approaches) help to recognize experiential knowledge and situate ethics in the service of enriched life experience. They also align with co-constructive approaches (e.g., participatory hermeneutic ethnographies) which help mobilize experiential knowledge toward effective change based on lived experiences (Montreuil and Carnevale 2018).

### Barriers to a living ethics stance

Although conveying aspirations of accessibility, personal and existential relevance, as well as inclusion and participation, we acknowledge that there are several barriers to the enactment of a living ethics stance.

*Power dynamics, hierarchies, and exclusion* are potent forces countering living ethics because living ethics is about the enforcing the capacities for individuals to live and ask questions about the moral aspects of their lives, while power and hierarchies tend to impose regimens of silence and taboos on the ability to express moral difficulties (e.g., restrictive policies on whistleblowing, effects of epistemic injustice (Fricker 2007)). Power provides the ability to deny the fallibility of knowledge by imposing certain knowledges and push back questions. Thus, creating space and time for ethical discussions necessarily raises questions about who is free to participate and to express themselves. Despite aspirations to openness, the milieu of health and healthcare–as we pointed out–is often constrained and constraining in this regard. Participatory and co-production approaches can help address these difficulties (Groot et al. 2022).

*Illiteracy and inaccessibility* are another important barrier to a living ethics because the ability to understand problems, vocabulary and discourses about them empowers people to participate in discussions while the opposite can inhibit participation. Vocabularies about health (e.g., in medicine, nursing, psychology, social work, physical therapy) are very technical, and require years of training to be mastered, which can render the understanding of health system even more difficult for patients and users of health services. Ethics risks falling prey to similar issues in the form of ethical jargon or in word traps (e.g., language about abilities and disabilities (Wolbring 2003)). Moreover, the issue of non-communicating patients (e.g., with disorders of consciousness or aphasia) or very young patients would call for further dedicated attention. Even if policies and directives ask to pay attention to these aspects of the communication, often they are not implemented and evaluated properly (Avard et al. 2010).

*Resources are limited* almost by their very nature, yet the recognition that ethics needs resources to live whether in the form of time, space, cultural values and mechanisms, personal energy, and intellectual ability to make sense of moral difficulties, communication, and dialogical resources, and so on, is less scrutinized. Ethics services are often under-resourced in terms of funding, human resources, and authority to make the case for the importance of human values in health (Guerrier 2006; Hurst et al. 2007; Machin and Wilkinson 2021) despite being considered as a valuable resource (Crico et al. 2021). In fact, the latter point is often part of the contention leading to moral tensions and conflicts because problems go unrecognized or are actively neglected in healthcare settings, leading to moral distress (Wilkinson 1988).

*Conformism and authority*, as we pointed out previously, are barriers to the creative freedom required for discussing what matters to a person because conformism is very much about obedience and compliance to norms and requirements imposed by authorities or societies. Ethics has tended to occupy regulatory spaces where it can transform into corporate compliance and undermine a substantive and meaningful account of ethics (Craze 2020). In the context of health, ethics is often equated with standards and guidelines, research ethics submission forms, and consultation forms. This stiffening of ethics can be detrimental to the ability for ethics to live. At the same time, the perspective of growth and flourishing fostered by living ethics may appear unrealistic as it may be wrongly interpreted as perfect mental health and wellbeing, but mental health and flourishing should be distinguished (Keller 2020) and flourishing is not a state of perfection or a nirvana removed from life circumstances.

*Polarized cultural, political, legal and social contexts* in health and politics–as noted before by Callahan (Callahan 2005)–also create difficulties for the expression of different and nuanced points of views as well as for open dialogue about moral aspects of health and health issues. Given how many liberal democracies work, wedge issues are effective strategies to divide public opinion and harvest votes and support (Peterson and Fayyad 2017). Health and ethics issues (e.g., gender-affirming care, abortion, stem cells, end-of-life issues) are no stranger to such strategic and divisive politics, which have amounted to “culture wars” in the USA (Hunter 1992) and now increasingly worldwide (Ipsos 2021). For example, polarization about COVID-19 vaccination, notably but not exclusively through social media, counterchecks the stance of a living ethics because ethics needs open and inclusive spaces of respectful dialogue and human relationships. Thus, current trends in the misuse of social media and related polarization and confrontational interactions are important challenges for a living ethics stance, but also potentially an opportunity for intervention.

Likewise, ongoing *misunderstandings about the nature of ethics* (e.g., as a form of morality, or as deontology), which would provide authoritative and final answers to moral questions, perpetuate longstanding confusion. In the context of health, the omnipresence of scientific knowledge and asymmetrical power relationships between users and providers of care and services amplifies the challenge of an open and inquiry-based form of ethics due to the effects of epistemic supremacy of biomedical science (Kidd and Carel 2017). Moreover, health is fraught with entrenched social and cultural understandings and beliefs which sometimes clash with evidence-based concepts of health and healthcare. Although *clinical ethics* has been less affected by–yet not immune to–trends toward evidence and the authoritative use of academic expertise (Engelhardt 2002), numerous analyses of *research ethics* have pointed to its highly restricted and bureaucratic nature which can undermine its meaningfulness (Trudel and Jean 2010, Cascio and Racine 2018). Likewise, efforts to self-regulate professions and produce contemporary deontology should not replace ethics per se. In summary, there is a constant challenge for ethics to resist commodification, and its simplification in various forms of professional deontologies and moralities.

## Future developments and deployment of a living ethics stance

We acknowledge that the initial proposal and description of a living ethics stance remains vague in certain respects such that more theoretical work will be needed to develop this idea from a conceptual standpoint. In order to tackle this, an international co-development workshop is already scheduled to take place where colleagues from North America, Europe, Australia, Africa, Latin America, and Asia will meet to exchange ideas on, for instance, conceptual and methodological aspects of living ethics. Moreover, the actual implications of a living ethics stance for different areas of health ethics will be the object of future publications which will notably explore the implications of a living ethics stance for (1) clinical and organizational ethics; (2) health policy and public health; (3) health ethics research and (4) earning and teaching health ethics. These topics were already part of the initial development of living ethics (see Fig. 1) but cannot be exposed in this first inaugural and programmatic paper. Looking forward, there is a need for ongoing dialogue about the nature of living ethics as a stance rather than an equivalent to traditional normative theory. Although we sidestepped this debate in this paper, living ethics could also be seen as a practice that contributes to the good and flourishing life because of the disposition it embodies and encourages. This issue was debated within our working group as some saw living ethics more strictly as a stance while others envisioned it as being a practice constitutive of the good life. Recent work on the nature of human flourishing reflects that flourishing is not only a state but a lifelong and ongoing process consistent with forms of pragmatist ethics. This idea aligns with a living ethics stance, which cultivate openness to ideas and experiences, fallibility, and ongoing moral learning. This stance may be part of a new generation of ethics theorizing which corresponds less to ideals of academic theory per se but more on the need and experience of theory users (e.g., Racine 2024a). However, at this stage, it is unclear if there are different forms of living ethics and what these are and could be since it is still very early in the development of this idea. For now, the idea of a stance reflects the current early stage of development of living ethics.

Methodologically, living ethics has strong acquaintances with participatory approaches as reflected in the affinities of the idea of living ethics with the idea of living theory (Whitehead and McNiff 2006) (see also section B of the online Supplementary Information). Indeed, once moral agents are envisioned as knowers and experimenters, then the appeal of participatory approaches becomes clear. This participatory orientation then calls for a certain account of moral agents and the role of ethicists and of ethical knowledge and expertise which does not correspond to the traditional and authoritative role of ethics theory and of ethicists (Abma et al. 2017; Metselaar et al. 2017; Inguaggiato et al. 2019). The methodological practices coherent with this orientation are in the realms of co-learning, dialogue, and facilitation which have been better defined in the area of clinical ethics (Metselaar et al. 2015; Inguaggiato et al. 2019, Inguaggiato, Widdershoven et al. 2021). However, there is still a need to define and evaluate these methodological practices in various domains of ethics (e.g., health, business, environment) and to understand and measure their impact since participatory methodologies, beyond being able to produce traditional outcomes, are also interested in capturing what is changed or learned through a process.

Finally, the practical relevance and impact of a living ethics stance will need to be established. The actual adoption and refinement of living ethics is currently a work in progress for various members of the working group. For example, a new five-year study funded by the Social Sciences and Humanities Research Council of Canada titled “Collaborative improvement of moral deliberation methods: An exercise in living ethics” is assembling a collective of 20 Canadian clinical ethicists to share on their ethics consultation practices, learn from each other’s practices, and trial advanced methods, all based on a participatory study design where participants interact as co-learners and thus genuine co-researchers. Another initiative, É-LABO, is a living lab initiated in 2022 aiming to create ethical spaces in clinical contexts using an adaptative and progressive participatory strategy to mobilize stakeholders (Racine et al. 2024). This project, funded by the Québec ministry of Economy, Innovation, and Energy is so far proving successful in unlocking conversations and raising awareness about moral problems embedded in two clinical practices settings involved in the project. It is still too soon to evaluate its complete multifaceted impact, but this is scheduled to take place in the form of participatory evaluation (Abma 2005; Abma and Widdershoven 2014). Finally, more recently, a newly funded larger scale laboratory for living ethics sponsored by the LRH Foundation is aiming to engage the personnel and service users of a research-intensive rehabilitation hospital to engage with ethical issues, again following a participatory and iterative research design (https://labolevier.com/). In the latter project, there is an explicit community orientation embedded in the goal of intervening and building on the ethics culture of the organization. These rapid and concrete developments are encouraging for the collective as we hope to grow collaborations and engagement with different theoretical, methodological, and practical implications of a living ethics stance.

## Conclusion

Health ethics has moved progressively from more theoretical to more engaged stances. As a collective of diverse health stakeholders, we observe how our own work is inspired by successive and progressive movement toward greater engagement, yet we aspire to make ethics a clearer and more powerful lever of human development and flourishing. Our effort of collective reflection led us to delineate a living ethics stance which describes a way of envisioning our work as being connected to moral life and also a facilitator of ongoing growth and flourishing. The aspiration of a living ethics is not to offer a new form of ethics (aka a normative ethics) but to support the adoption of a different stance toward moral problems as well as toward ethics theory and ethics practice. We acknowledge that this stance cannot be reduced to a few adjectives; instead, it enacts a more profound change toward user-orientation, moral experience and existence, human flourishing, and the role of ethics in helping people become active and empowered agents of their own moral lives. The idea of a living ethics stance has numerous theoretical, methodological, and practical implications which this programmatic paper also laid out, although more will be needed to refine and advance the idea as it is being enacted. Already, there are encouraging signs that this effort is paving the way to various concrete innovative and creative exercises. We hope that our experience is both informative about the nature of a living ethics stance and invites further participatory efforts of local and international collectives of stakeholders to reflect on the foundations and orientation of their work and aspirations.

### Supplementary Information

Below is the link to the electronic supplementary material.

Supplementary file 1 (PDF 47 kb)

## Data Availability

There are no data associated with this manuscript which is a discussion paper. Please contact eric.racine@ircm.qc.ca for any data-related requests.

## Footnotes

1 Although often undistinguished and etymologically synonymous terms derived from Latin and Greek respectively, we use the term moral and morality to designate the more implicit habits which structure and guide our lives while we reserve the term of ethics to designate the structured and explicit reflection on moral habits and moral life. Hence, ethics takes moral experience and moral life as its object of inquiry and is thus a science of this domain of human life.

2 We tend to use the concept of bioethics to describe a certain kind of health ethics which has emerged initially in North America (Doucet 1996) such that the use of health ethics is broader.

3 Without elaborating and discussing the specific history of Québec, and the development of health ethics therein, we can simply underscore that it represents a different historiographic, political, legal, national, cultural and linguistic environment to other provinces or states part of the Canadian federation and of North America more generally (see notably (Lamonde 2019)). Québec is home to some of the early activities in bioethics such as at the Bioethics Centre of the Institut de recherches cliniques de Montréal starting in 1976. It now counts several training programs and research groups.

4 We use the notion of human flourishing to designate the focus of ethics and as a contemporary rendition of Eudaimonia (Ryff and Singer 2008). It is beyond the scope of this paper to engage with the elaborate and extensive literature on this topic but human flourishing signals the engagement of ethics with the idea of making the world a better place to live. Our use of this concept is open; it encourages ongoing learning about what a flourishing life is (Lanteigne, Genest and Racine 2021; Willen 2022).

5 We note – thanks to one of the reviewers–that the notion of living ethics has been used before in Russian philosophy, notably by Nicolas Roerich, a painter and spiritualist (Gindilis and Frolov 2002). In this context, Zhivaya Etika (Живaя Этикa, translated as living ethics in English) designates Agni Yoga, a form of theosophy about cosmic order, but these are quite different than our use and context inspired by pragmatism. Additionally, a textbook by Russ Shafer-Landau bears the name “Living ethics: An introduction with readings” (Shafer-Landau 2021) but the words are used simply descriptively and do not point to a specific approach or stance.

6 In fact, we are likely to healthily disagree amongst ourselves like our fellow citizens about the several ways in which specific theories and principles should be used and what are the appropriate tools to wield in the face of specific problems.

7 Although academic efforts have long targeted the laudable development of ethics theories, concepts, and principles, further work on stances in ethics as undertaken in this paper, could make embodied and embedded assumptions more explicit. Doing this could help: (1) provide a clearer direction, an orientation, a vision for ethics; (2) help guide and enlighten ethics practice consistent with the stance adopted; (3) serve as a source of projections and hypotheses about what should be pursued academically and practically; (4) provide a milestone or a framework for the evaluation of practices; (5) reveal gaps and divergences about different views of ethics; (6) make ideas more accessible and amenable to discussion and revision.

8 In *Brainstorm*, Dennett (1981) differentiates different stances which can be taken in the understanding of the human mind: (1) a physical stance, (2) a design stance, and an (3) intentional stance. These stances are different points of views with different purposes and involve the use of different kinds of knowledge (e.g., physics-chemistry, biology-engineering, psychology, respectively).

9 In pragmatist theory, transaction does not designate a relationship with economic or superficial contractual overtones. Rather, it means that human beings are in constant exchange with their environments and these exchanges are based on actions, thus they are trans-actions (Brinkmann 2011, Foucart 2012/3). It is reminiscent of the comprehensive account of relationships offered by feminist scholars (Barclay 2000, Sullivan 2001).

10 As we discuss below, narratives are powerful ways by which the meaning of experiences are conveyed because they allow people to tell how certain events ‘fit’ into one’s life and their impact therein.

## Declarations

### Human and animal participants

There are no human subject, human tissue samples or animal data in this manuscript which is a discussion paper.
