# Supplementary material for: Living ethics: a stance and its implications in health ethics
Source: Med Health Care Philos. 2024 Mar 13;27(2):137–54. doi: 10.1007/s11019-024-10197-9 (PMC11076378; doi:10.1007/s11019-024-10197-9)
Supplement: Supplementary file 1 — (PDF 47 kb) [file 11019_2024_10197_MOESM1_ESM.pdf]

SUPPLEMENTARY INFORMATION (ONLINE-ONLY MATERIAL)

A. SUPPLEMENTAL INFORMATION: METHODS AND PROCESS

To lay out the concept of a living ethics stance, we followed a collaborative and participatory process known as instrumentalist concept analysis (Racine et al. 2019). This concept development and participatory enrichment method designates a process by which ethics concepts are first documented, notably according to their goals as instruments (i.e., function identification). Once identified, their functions (or goals) are then enriched based on stakeholder consultations and eventually tested in the light of experience, leading to further iterative enrichment. In this process, ethics concepts are never ends in themselves but rather means through which human experience is enriched to further human flourishing. Accordingly, concepts can thus be assessed for their ability to enact meaningful purposes. Therefore, instrumentalist analysis is a process of iterative co-creation, i.e., participatory development through subsequent cycles in which stakeholders are engaged in the process. Implicit or explicit stances about what ethics should achieve and why, remain a source of heated debate and consequently merit attention – notably through dedicated instrumentalist analysis–in the development of more concerted and collaborative efforts in contexts such as health ethics but also in other settings (e.g., business, environment, etc.).

Initial informal local consultations were undertaken by the first lead author as part of a deliberate effort to enrich local collaborations and to think about the contribution of a roughly but not exclusively pragmatist account of ethics to local practices and scholarship based on existing and ongoing research as well as networking activities. As part of these initial consultations, clinicians actively involved in ethics, patient partners, professional ethicists coming from various

settings, academic ethicists, other scholars in health and social sciences, and practitioners were presented with the opportunity of co-developing a program of research to further ethics theory and methodology as an instrument to support human flourishing through both informal and formal presentations. From these conversations emerged the project of conceptualizing a form of living ethics and related practices which would further existing commitments and collaborations to make ethics a clearer and more powerful lever of human development and wellbeing. At the very heart of this project was the desire to share different perspectives about the nature of ethics as well as its potential for various practices and wide-ranging expertise, disciplinary training, and domains of activity.

Following these consultations, an exploratory literature review was undertaken during the summer of 2021 to examine relevant and neighboring ideas and views about ethics, and their affinities with an initial rough concept of living ethics. This helped us further situate our proposal and clarify its connections to existing scholarship and practices. We recognized from the onset that the concept of a living ethics stance has strong affinities with participatory action research as developed in the context of education, where the idea of living theory has been formulated (Whitehead and McNiff 2006). Likewise, various initiatives, notably in Northern Europe, have articulated and explored similar proposals for dialogical ethics (Widdershoven, Abma, and Molewijk 2009), participatory bioethics (Abma, Voskes, and Widdershoven 2017; Metselaar et al. 2017), hermeneutic and deliberative ethics (Widdershoven, Abma, and Molewijk 2009; Gutmann and Thompson 1997; Abma, 2014), including overlaps with pragmatism (Maesschalck 2011; Hartman et al. 2019; Inguaggiato, Widdershoven, and Metselaar 2021). This scholarship aligns with like-minded local initiatives and developments in Québec such as deliberative and dialogical ethics (Doucet 1994; Doucet, Larouche, and Melchin 2001; Doucet et al. 2007), pragmatist ethics

(Racine 2010, 2008), and participatory hermeneutical ethics research (Montreuil et al. 2017; Montreuil and Carnevale 2018).

Based on the initial informal consultations, follow-up invitations were sent during the summer of 2021 to colleagues who were consulted informally previously to join a working group on the exploration of a living ethics (see Figure 1). All colleagues were offered to join as co-researchers and co-authors of this writing project. The final recruitment process led to the current list of co-authors, although along the way, some potential co-authors decided to limit their participation and are simply acknowledged.

The working group held a total of 8 workshop-like meetings (see Figure 1), and followed the structure and process of other similar large working group writing, reflection, and participatory ethics guidance development projects (Racine et al. 2017; Racine et al. 2014; Sample et al. 2019; Cascio, Weiss, and Racine 2020). Meetings were chaired by the first lead author, seconded by the second lead author, and supported by the prior reading and revision of a draft in progress, i.e., a "living" document accessible through an online sharing platform. Each 90-minute meeting was supported by preceding updates to the work document, generally two weeks before the meeting. Meetings were held in French and typically started with short updates (except for the first meeting, when the project and its process were presented) about the progression of the writing and reflection projects by the first lead author, followed by about an hour-long series of exchanges between all members of the working group through open discussion regarding different aspects of the proposal. Meetings were video-recorded and professionally transcribed to help further ongoing reflections and writing. Meeting recordings and transcripts were made available to the working group members as well as all other supporting project documents on a shared online platform. Following these meetings, the working group's comments and changes to the document written in English

were reconciled and advanced by the lead author with the help of the second lead author (e.g., drafting syntheses based on meeting transcripts), followed by a two-week period of online open commenting and co-writing by working group members.

The working group decided to establish four subgroups (clinical and organizational ethics, health policy and public health, health ethics research, learning and teaching health ethics) to further the working group's reflections in specific domains of health ethics activity. Leaders of these subgroups were selected based on personal interest, on their expertise, and their availability to lead subgroup meetings. Subgroup leaders were provided with a moderation guide structured around different topics to further explore and illustrate experiences of living ethics as well as its aspirational goals, in their specific domain of activity, with their subgroup colleagues. These discussions were guided by the following questions: (1) *Identify the characteristics and orientations of a living ethics (based on two initial meanings and their attributes but not only restricted to these*[1]*) in your 'field'.* (2) *What are concrete examples of living ethics practice? What are counter-examples?* (3) *How is a living ethics embodied in the tasks of an ethicist?* (4) *What changes does a 'living ethics' orientation support or foster and what significant progress would be needed?* (5) *What would be examples of a living ethics project in your 'field'?* (6) *What are the limits of living ethics in your 'field'?* There were two meetings dedicated to discussion in the subgroups. Writing progressed, as subgroup meetings were transcribed and analyzed, to identify commonalities and differences across domains of health ethics represented in the subgroups.

To support the working group, an international advisory committee was constituted. We recruited experts reputed for having developed neighboring ideas from which we drew inspiration

[1] These two initial meanings are explained in the paper (Fig. 2) and represented the starting point of the working group's formal deliberation and writing process.

and with whom we could also foresee international-level collaborations and exchanges based on our common scholarly knowledge or professional networks. Advisory committee members were consulted on the penultimate version of the manuscript, which included all key sections of the manuscript. Advisory committee members' comments were collected over a pre-established 2-week period and compiled by the first and second lead authors in table format, shared as part of the working document. Finally discussed in a plenary session with all the members of the working group to prepare the final version of the manuscript.

In writing this paper, we pulled from our knowledge of the literature and the insights generated by the working group to provide a more coherent account of living ethics and make sense of this view as it relates to our work and the tasks we accomplish in ethics. Such a community practice-orientation to health ethics development – rather than an individual approach – guided the project. Reflection on a stance, like a living ethics, can hardly rely on single-handed views but rather can advance within extensive collaboration and enrichment through mutual learning.

B. SUPPLEMENTAL INFORMATION: BACKGROUND SUPPORT FOR LIVING ETHICS

Living ethics builds from both past and current scholarship. Since extensive background information cannot be detailed in the initial programmatic discussion paper, instead we briefly describe how a living ethics stance is supported by both (1) classic pragmatism and living theory as well as (2) contemporary scholarship.

Support for a living ethics stance in classic pragmatism and living theory

Living ethics – as used in this paper – is a rather recent term, but there are important precedents that have set the stage for the evolution toward this stance. In our own work, the development of a living ethics stance is partly inspired by an understanding of pragmatism that takes at heart the view that "[p]ragmatism is a philosophical tradition – that very broadly – understands knowing the world as inseparable from agency within it" (Legg and Hookway 2019). In this light, ethics – insofar as it is both a form of knowledge and practice – is thus an attempt to understand and direct human morality in ways that are open to others and to life experiences. Moreover, ethics facilitate the enactment of values which contribute to growth and flourishing (Dewey 1922). Living ethics also builds from the idea that the meaning of human existence, being central to ethics, can be partly only captured with minimal and procedural forms of ethics (Williams 1985). Ethics also calls for an engagement with substantive eudemonistic concepts. John Dewey, for instance, suggested that the true categorical imperative – although he recused the universalistic nature of such a formulation – was to "…act as to increase the meaning of present experience" (Dewey cited in (Pekarsky 1990)). Living ethics is also freely inspired by Jack Whitehead's concept of living theory, developed in the context of education research (Whitehead

and McNiff 2006), and by certain views of action research as a living practice (Carson and Sumara 1997).[2]

Accordingly, pulling from these inspirations, a living ethics and more precisely a living approach or stance to ethics must facilitate the connection of ethics to actual human life, human existence, and human experience, reminiscent of the scholarship on relational autonomy (Mackenzie and Stoljar 2000) and contextualized autonomy (Racine et al. 2021). It must also position ethics as a facilitator of human moral growth as well as moral meaning and significance in ways that are concrete and reportable. This should be done in a way that is accessible (e.g., in plain language and connected to everyday experience) and in which the traditional *teaching* perspective of ethics is embedded in a richer and more engaging *learning* perspective.[3] Such a lifelong experiential learning perspective (Kolb 1984; Miettinen 2000) is supported by a living ethics stance because our knowledge about ourselves and the world we live in is intrinsically incomplete, limited, and always liable to revision. A living ethics stance thus incorporates the idea that knowledge, including ethical knowledge, evolves.[4]

A living ethics stance also furthers previous developments in educational science (Whitehead and McNiff 2006) and in qualitative research (Carson and Sumara 1997) where agents,

[2] These contributions stress the nature of participatory research as a living and evolving practice where acquiring knowledge is recognized as being an action on the world and a way of interacting with the world. Importantly, they also stress how knowledge benefits from being connected to experience and concrete tasks.

[3] For a discussion on the role of ethics in co-learning and co-creation and of ethicists as facilitators or mediators, see notably (Inguaggiato et al. 2019; Metselaar, Molewijk, and Widdershoven 2015).

[4] This is also why participation is almost synonymous with living because living beings continuously transact with their environments (Brinkmann 2011; Sullivan 2001). Biological or social death occur when physical (e.g., breathing) (Baker and Shemie 2014; Bernat 2002; Shemie et al. 2014) or social (e.g., isolation and marginalization) transactions cease (Patterson 1985; Card 2003). Transactions in the pragmatist sense are always limited or restricted to certain environments and to certain people. The ability of different people to transact is also dependent on several facilitating and impeding factors (e.g., socioeconomic status, literacy). Thus, participation represents a form of transaction where a moral agent is able to enact important values such that they actualize themselves (Racine 2024).

such as teachers, are invited to develop living theories of their own educational activities. A noteworthy addition in the context of ethics is that Dewey advanced the notion that ethics theory needed to be a living and adaptive instrument in service of growth and human flourishing (Dewey 1922). He remarked that: “In fact situations into which change and the unexpected enter are a challenge to intelligence to create new principles. Morals must be a growing science if it is to be a science at all, not merely because all truth has not yet been appropriated by the mind of man [human beings], but because life is a moving in which old moral truth ceases to apply” (Dewey 1922). Another important inspiration for a living ethics stance is Kolb’s model of experiential learning (Kolb 1984), a model shaped by pragmatist insights (Miettinen 2000). Moreover, some of the ideas advanced in pragmatism, education research, and qualitative research resonate with recent calls for participatory bioethics (Abma, Voskes, and Widdershoven 2017; Metselaar et al. 2017), the capability approach (Clark, Biggeri, and Frediani 2019), and pragmatist-hermeneutic approaches in bioethics (Widdershoven, Abma, and Molewijk 2009; Montreuil and Carnevale 2018) as developed notably in the method of moral case deliberation (Weidema et al. 2013). Dewey’s insight about the living nature of ethics theory is one that we build from as well as contemporary scholarship on human flourishing, participatory research epistemologies, and participatory bioethics.

Support for a living ethics stance in contemporary scholarship

Contemporary scholarship in bioethics – and in other areas of ethics – has heralded some of the insights encapsulated in living ethics such that the current proposals build on them. For example, current research in psychology and social science strongly supports the view that active participation to one’s shaping of their life and their society is essential to living a flourishing and satisfying life (Clark, Biggeri, and Frediani 2019; Racine 2024). Accordingly, in this light, ethics

can be re-described as the process by which human beings, individually and collectively, are empowered to participate in their own development and take hold of their futures in ways that foster human development and flourishing. Such an account of ethics corresponds to a living ethics stance as we describe it. It provides footing for an account of ethics as a life project, a way of relating to the moral dimension of human existence. It also fosters an open and learning perspective on what is most valuable in human existence and seeks ways of bringing it about.

A living ethics also integrates insights from research showing connections between openness in life (what psychologists call open-mindedness) and the willingness to learn, the ability to find meaning and pursue a meaningful life, active involvement in one's existence as an autonomously oriented life as well as in nurturing positive and nourishing relationships with others (Ryff 2014). Therein, there is an intimate connection between the well-being of others and the ability for someone to pursue a flourishing life (Ryff 2014). A living ethics stance, because of its emphasis on the connection between lived experience and living theory (see Fig. 2), is a way to bridge the growing body of evidence about the importance of intrinsically motivated behavior (Deci and Ryan 1985, 2000; Ryan and Deci 2017) with the long-described eudemonistic goal of the good life or the life worth living, i.e., *eudaimonia* (Chambliss 1993; Fahey 2010), thus reconnecting with ancient strands of ethics theory (Parry 2021). Accordingly, we can look to experience to test what James called the "cash value of our ideas" (Cotkin 1985) in the context of human morality. Indeed, ethics scholarship and practice, if worthy, speak to actual lived experience and contributes to its enrichment in some shape or form (Pekarsky 1990). Existential and experiential knowledge is not knowledge which can be instilled mechanically or generated single-handedly (Kolb 1984), it must rely on favorable contexts in which questioning, open dialogue and the sharing of difficult life experiences is rendered possible and acceptable (Lacroix et al. 2017;

Legault 1999). As discussed below, it is often best communicated and explored using narrative or artistic individual or collective forms of expression. This not only allows for the explanation of events but also conveys the interpretation and meaning of lived experiences (Polkinghorne 1988; Denborough 2008) as well as the embodied aspects of lived experience (Adra et al. 2021).

REFERENCES (NOT FOUND IN THE MANUSCRIPT)

Abma, Tineke A., Yolande Voskes, and Guy Widdershoven. 2017. Participatory bioethics research and its social impact: The case of coercion reduction in psychiatry. *Bioethics* 31(2): 144-52. https://doi.org/10.1111/bioe.12319

Adra, Hoda, et al. *Voices of the Nakba: A living history of Palestine*, ed. Diana Allan. London: Pluto Press.

Baker, Andrew, and Sam D. Shemie. 2014. Biophilosophical basis for identifying the death of a person. *Journal of Critical Care* 29(4): 687-89. https://doi.org/10.1016/j.jcrc.2014.04.013

Bernat, James L. 2002. The biophilosophical basis of whole-brain death. *Social Philosophy & Policy* 19(2): 324-42. https://doi.org/10.1017/S0265052502192132

Brinkmann, Svend. 2011. Dewey's neglected psychology: Rediscovering his transactional approach. *Theory & Psychology*. 21(3): 298-317. https://doi.org/10.1177/0959354310376

Card, Claudia. 2003. Genocide and social death. *Hypatia* 18(1): 63-79.

Carson, Terrance R., and Dennis J. Sumara. 1997. *Action research as a living practice*. Ann Arbor, MI: University of Michigan/Peter Lang.

Chambliss, J. J. 1993. Common ground in Aristotle's and Dewey's theories of conduct. *Educational Theory* 43: 249-60. https://doi.org/10.1111/j.1741-5446.1993.00249.x

Clark, David A., Mario Biggeri, and Alexandre A. Frediani. 2019. *The capability approach, empowerment and participation: Concepts, methods and applications*. London: Palgrave-Macmillan.

Cotkin, George. 1985. William James and the cash-value metaphor. *ETC: A Review of General Semantics* 42(1): 37-46.

Edward L. Deci and Richard M. Ryan. 2000. The "what" and "why" of goal pursuits: Human needs and self-determination theory in human behavior. *Psychological Inquiry* 11(4): 227-68. https://doi.org/10.1207/S15327965PLI1104_01

Doucet, Hubert. 1994. Le développement des morales, des législations et des codes, garder le dialogue ouvert et la conscience inquiète. In Office des personnes handicapées du Québec (ed.), *Élargir les horizons: Perspectives scientifiques sur l'intégration sociale*. Québec: Éditions Multimondes, 135-141.

Doucet, Hubert , Jean-Marc Larouche, and Kenneth R. Melchin. 2001. *Ethical deliberation in multiprofessional health care teams*. Ottawa: University of Ottawa Press.

Doucet, Hubert, Marianne Dion-Labrie, Céline Durand, and Isabelle Boutin-Ganache. 2007. Genomics and modes for democratic dialogue: An analysis of two projects. In Bartha M. Knoppers (ed.), *Genomics and public health: Legal and socio-ethical perspectives*. Leiden: Martinus Nijhoff International, 287-304.

Fahey, Gregory M. 2010. The idea of the good in John Dewey and Aristotle. *Pragmatism and Neopragmatism* 3(2): 201-26. https://doi.org/10.5840/eip2002328

Gutmann, Amy, and Dennis Thompson. 1997. Deliberating about bioethics. *Hastings Center Report*, 27(3): 38-41. https://doi.org/10.2307/3528667

Hartman, Laura, Suzanne Metselaar, Guy Widdershoven, and Bert Molewijk. 2019. Developing a 'moral compass tool' based on moral case deliberations: A pragmatic hermeneutic approach to clinical ethics. *Bioethics* 33(9): 1012-21. https://doi.org/10.1111/bioe.12617

Kolb, David A. 1984. *Experiential learning*. Englewood Cliffs, NJ: Prentice Hall.

Lacroix, André, Allison Marchildon, Luc Bégin, and Marie-Claude Boudreau. 2017. *Former à l'éthique en organisation - Une approche pragmatiste*. Québec: Presses de l'Université du Québec.

Legault, Georges-Auguste. 1999. *Professionnalisme et délibération éthique: manuel d'aide à la décision responsable*. Québec: Presses de l'Université du Québec.

Legg, Catherine, and Christopher Hookway. 2019. Pragmatism. Stanford Encyclopedia of Philosophy. https://plato.stanford.edu/entries/pragmatism/. Accessed January 31 2020.

Maesschalck, M. Marc. 2011. *Transformations de l'éthique*. Bruxelles: Peter Lang Verlag.

Miettinen, Reijo. 2000. The concept of experiential learning and John Dewey's theory of reflective thought and action. *International Journal of Lifelong Education* 19(1): 54-72. https://doi.org/10.1080/026013700293458

Patterson, Orlando. 1985. *Slavery and social death: A comparative study*. Cambridge: Harvard University Press.

Pekarsky, Daniel. 1990. Dewey's conception of growth reconsidered. *Educational Theory* 40: 283-94. https://doi.org/10.1111/j.1741-5446.1990.00283

Polkinghorne, Donald. 1988. *Narrative knowing and the human sciences*. Albany: SUNY Press.

Racine, Eric. 2008. Which naturalism for bioethics? A defense of moderate (pragmatic) naturalism. *Bioethics* 22(2): 92-100. https://doi.org/10.1111/j.1467-8519.2007.00604

Racine, Eric, Emily Bell, Allison Yan, Gail Andrew, Lorraine E. Bell, Margaret Clarke, Veljko Dubljevic, Dan Goldowitz, Annie Janvier, Kaitlyn McLachlan, Nazeem Muhajarine, David Nicholas, Maryam Oskoui, Carmen Rasmussen, Lisa A. Rasmussen, Wendy Roberts, Michael Shevell, Lucie Wade, and Jerome Y. Yager. 2014. Ethics challenges of transition from paediatric to adult health care services for young adults with neurodevelopmental disabilities. *Paediatrics & Child Health* 19(2): 65-8. https://doi.org/10.1093/pch/19.2.65

Racine, Eric, Ariel Cascio, Marjorie Montreuil, and Aline Bogossian. 2019. Instrumentalist analyses of the functions of ethics concepts: A proposal to bridge conceptual and empirical ethics methodology. *Theoretical Medicine and Bioethics* 40(4): 253–78. https://doi.org/10.1007/s11017-019-09502-y

Ryan, Richard M. and Edward L. Deci (2017). *Self-determination theory: Basic psychological needs in motivation, development, and wellness*. New York: Guilford Publishing.

Ryff, Carol D. 2014. Psychological well-being revisited: Advances in the science and practice of eudaimonia. *Psychotherapy and Psychosomatics* 83(1): 10-28. https://doi.org/10.1159/000353263

Sample, Matthew, Sebastian Sattler, David Rodriguez-Arias, Stefanie Blain-Moraes, and Eric Racine. 2019. Do publics share experts' concerns about brain–computer interfaces? A trinational survey on the ethics of neural technologies. *Science, Technology, and Human Values* 45(6): 1242-1270. https://doi.org/10.1177/01622439198792

Shemie, Sam D., Laura Hornby, Andrew Baker, Jeanne Teitelbaum, Sylvia Torrance, Kimberly Young, Alexander M. Capron, James L. Bernat, and Luc Noel. 2014. International guideline development for the determination of death. *Intensive Care Medicine* 40(6): 788-97. https://doi.org/10.1007/s00134-014-3242-7

Weidema, Froukje. C., Bert Molewijk, Frans Kamsteeg, and Guy Widdershoven. 2013. Aims and harvest of moral case deliberation. *Nursing Ethics* 20(6): 617-31. https://doi.org/10.1177/09697330124737

Williams, Bernard. 1985. *Ethics and the limits of philosophy*. London: Fontana Press/Collins.
